# Solution NMR Backbone Assignment of the C-Terminal Region of Human Dynein Light Intermediate Chain 2 (LIC2-C) Unveils Structural Resemblance with Its Homologue LIC1-C

**DOI:** 10.3390/magnetochemistry9070166

**Published:** 2023-06-28

**Authors:** Morkos A. Henen, Natasia Paukovich, Rytis Prekeris, Beat Vögeli

**Affiliations:** 1 Department of Biochemistry and Molecular Genetics, School of Medicine, University of Colorado Anschutz Medical Campus, Aurora, CO 80045, USA;; 2 Department of Cell and Developmental Biology, School of Medicine, University of Colorado Anschutz Medical Campus, Aurora, CO 80045, USA;

**Keywords:** human dynein, light intermediate chain, LIC2, SSP

## Abstract

Dynein, a homodimeric protein complex, plays a pivotal role in retrograde transportation along microtubules within cells. It consists of various subunits, among which the light intermediate chain (LIC) performs diverse functions, including cargo adaptor binding. In contrast to the vertebrate LIC homolog LIC1, LIC2 has received relatively limited characterization thus far, despite partially orthogonal functional roles. In this study, we present a near-to-complete backbone NMR chemical shift assignment of the C-terminal region of the light intermediate chain 2 of human dynein 1 (LIC2-C). We perform a comparative analysis of the secondary structure propensity of LIC2-C with the one previously reported for LIC1-C and show that the two transient helices in LIC1 that interact with motor adaptors are also present in LIC2.

## 1. Biological Context

Dynein 1 (‘dynein’), one of the two classes of dynein in vertebrates, is the predominant microtubule minus-end-directed motor [1,2]. Dynein is actively involved in a diverse range of cellular trafficking processes, including the cargo transport of proteins, RNA and vesicles, nuclear migration, or cell division [3,4]. Processive movement of dynein on microtubules requires binding to the cofactor dynactin, and specific adaptor proteins link the dynein–dynactin complex to cargo [5].

Dynein is a homodimeric multi-protein dynein complex, composed of two heavy chains and multiple accessory chains consisting of intermediate, light, and light intermediate chains [6]. The light intermediate chain (LIC) binds the various adaptors and has been identified as one factor responsible for metaphase to anaphase progression through the inactivation of the spindle assembly checkpoint (SAC) [7]. The close homologs LIC1 and LIC2 are believed to work in mutually exclusive complexes to perform distinct [8,9] but also compensatory functions [10]. LIC1 plays a larger role in maintaining spindle pole integrity. In contrast, LIC2 has been reported to govern spindle orientation through interactions with 14–3-3, Par3, and NuMA [11]. Notably, LIC2, but not LIC1, has been found to transport NuMA asymmetrically to the spindle poles [11]. The structural basis of this disparity between LIC1 and LIC2 is not known. While we have studied LIC1 in solution at the structural level [12], there is no such data on LIC2 available.

**Figure 1.**
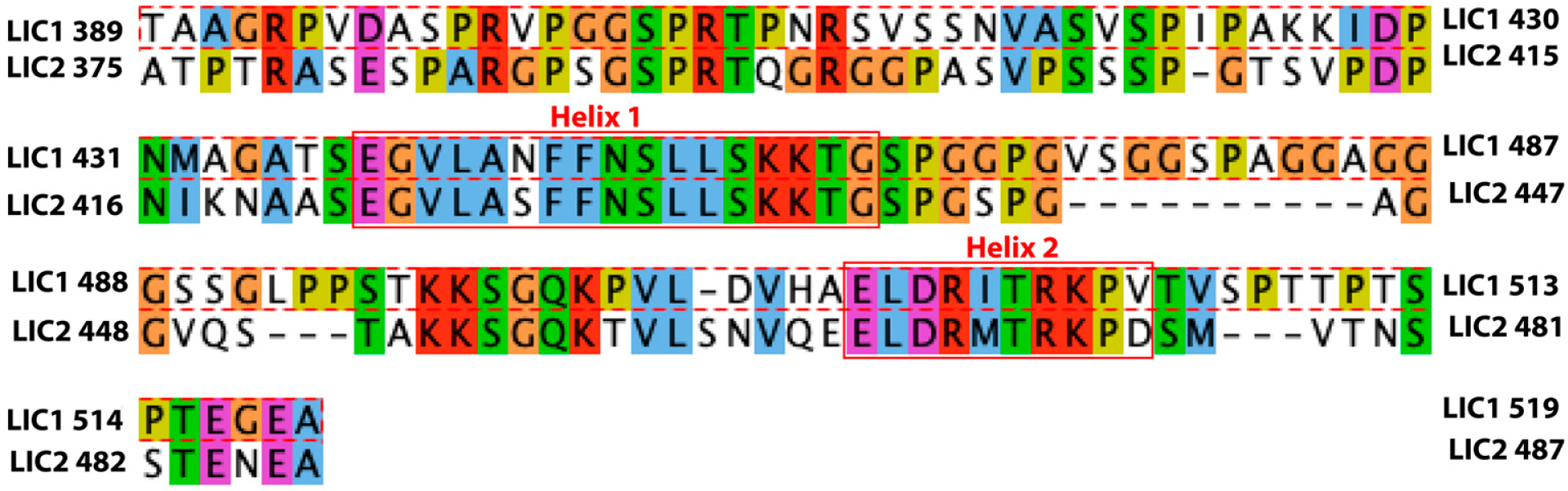
Sequence alignment between LIC1-C (residues 381–523) and LIC2-C (375–492). These regions exhibit ~47% sequence similarity, but the regions harboring helix 1 and helix 2 in LIC1-C are highly conserved in LIC2-C. Residue colors indicate side-chain chemistry: yellow, proline; blue, hydrophobic; red, positively charged; magenta, negatively charged; green, polar uncharged; orange, glycine; cyan, aromatic.

Employing NMR chemical shifts, we have previously shown that the C-terminal region of LIC1 is disordered with a propensity to form two helices (helices 1 and 2) [12]. Our NMR and animal model data demonstrated that helix 1 is essential for binding all tested dynein adaptors, and helix 2 plays an additional but non-essential role. Pulldown assays indicated that LIC2-C also binds to these adaptors, but we did not pursue a more detailed characterization, such as an NMR analysis. Sequence alignment between the C-terminal regions of LIC1 and LIC2 reveals that the two regions which comprise helical propensity in LIC1-C are conserved in LIC2-C (Figure 1).

A comprehensive understanding of LIC2-C’s mechanism underlying intracellular transport and specific differences with LIC1 is critical to rationalize the implications of dysfunctions and mutations in various diseases [13,14]. In this article, we take the first steps towards the characterization of the C-terminal region of LIC2-C using NMR, by assigning the chemical shifts of the backbone and deciphering secondary structure features.

## 2. Methods and Experiments

### 2.1. Protein Expression and Purification

#### (A) Construction Design and Cloning:

To obtain the LIC2-C construct (residues 375–492 of LIC2), we introduced an additional tryptophan residue at the N-terminus for convenient measurement of protein concentration using absorbance at 280 nm. Furthermore, a C-terminal (6x::His) tag was incorporated. The construct was successfully cloned into the pGEX-6p-1 expression vector, which contained an N-terminal GSTag fused to a preScission protease cleavage site. The plasmid was transformed into Escherichia coli strain BL21 (LEMO21-DE3).

#### (B) Media Preparation and Induction:

For protein expression, M9 media was prepared, containing 1 g/L ^15^N-ammonium chloride and 2 g/L ^13^C-glucose. The expression was induced with 0.4 mM isopropyl-1-thio-d-galactopyranoside when the optical density at 600 nm (A600) of the culture reached 0.6. The induced culture was incubated overnight at 20 °C.

#### (C) Cell Harvesting and Lysis:

The cells were harvested by centrifugation at 4 °C for 10 min at 5000× *g*. Subsequently, the cell pellet was resuspended in low-imidazole binding buffer (20 mM HEPES, 200 mM NaCl, 1 mM EGTA, 1 mM MgCl_2_, 1 mM NaN_3_, 20 mM imidazole, pH 7.3), subjected to sonication for cell disruption, and the lysate was clarified by centrifugation at 30,900× *g*.

#### (D) Protein Purification:

The clarified lysate was purified using a HisTrap FF column (Cytiva). The protein of interest was eluted using high-imidazole buffer (20 mM HEPES, 200 mM NaCl, 1 mM EGTA, 1 mM MgCl_2_, 1 mM NaN_3_, 200 mM imidazole, pH 7.3). Subsequently, the eluted protein was dialyzed in preScission protease cleavage buffer (50 mM Tris-HCl, 150 mM NaCl, 10 mM EDTA, 1 mM DTT, pH 8.0, 20% glycerol). The protein was incubated with the protease overnight at 4 °C, and the cleavage efficiency was assessed by analyzing the cleaved product on a 4–12% gradient SDS-PAGE gel.

The cleaved protein was concentrated to a final volume of 4 mL. Further purification was performed using a size-exclusion HiLoad 16/600 Superdex 75 pg column (Cytiva) in NMR buffer (50 mM NaP, 150 mM NaCl, 1 mM DTT, 0.02% NaN_3_, pH 6.5). The relevant fractions were concentrated using a 3000 MWCO concentrator to achieve a final concentration of 840 μM for subsequent NMR measurements.

### 2.2. NMR Spectroscopy

^13^C- and ^15^N-labeled NMR sample of LIC2 was prepared in the NMR buffer at a concentration of 840 μM and measured in a 5 mm Shigemi tube. NMR spectra were acquired on BRUKER Avance NEO 600 MHz triple-resonance cryoprobe spectrometers at 25 °C. Backbone assignment was accomplished using ^1^H-^15^N HSQC, 3D HNCACB, HN(co)CACB [15,16], and HNN pulse sequences [17]. The 3D spectra were obtained using a nonuniform sampling (NUS) scheme generated by the NUS@HMS scheme generator [18]. In the direct dimension, 2048 complex data points were acquired, while the indirect ^13^C and ^15^N dimensions were subsampled by 25–30% from the original 256 and 92 points, respectively. The spectral widths used for all experiments were 8196 Hz (^1^H), 2129 Hz (^15^N), and 12,076 Hz (13Cα/13Cβ). The number of scans was set to 16 and 32 for 3D experiments and 2D HSQC, respectively, with an interscan delay of 1.0 s. Reconstruction of the 3D NUS spectra was performed using the hmsIST software [18], while linearly acquired 2D spectra were zero-filled using NUS as an alternative to linear prediction. A solvent subtraction function was applied in the direct dimension. Further data processing and visualization were conducted using NMRpipe/NMRDraw [19] and NMRFAM Sparky [20]. The resonance assignment was carried out using the CCPNmr analysis software v2.5.1 [21].

## 3. Assignment and Data Deposition

**Figure 2.**
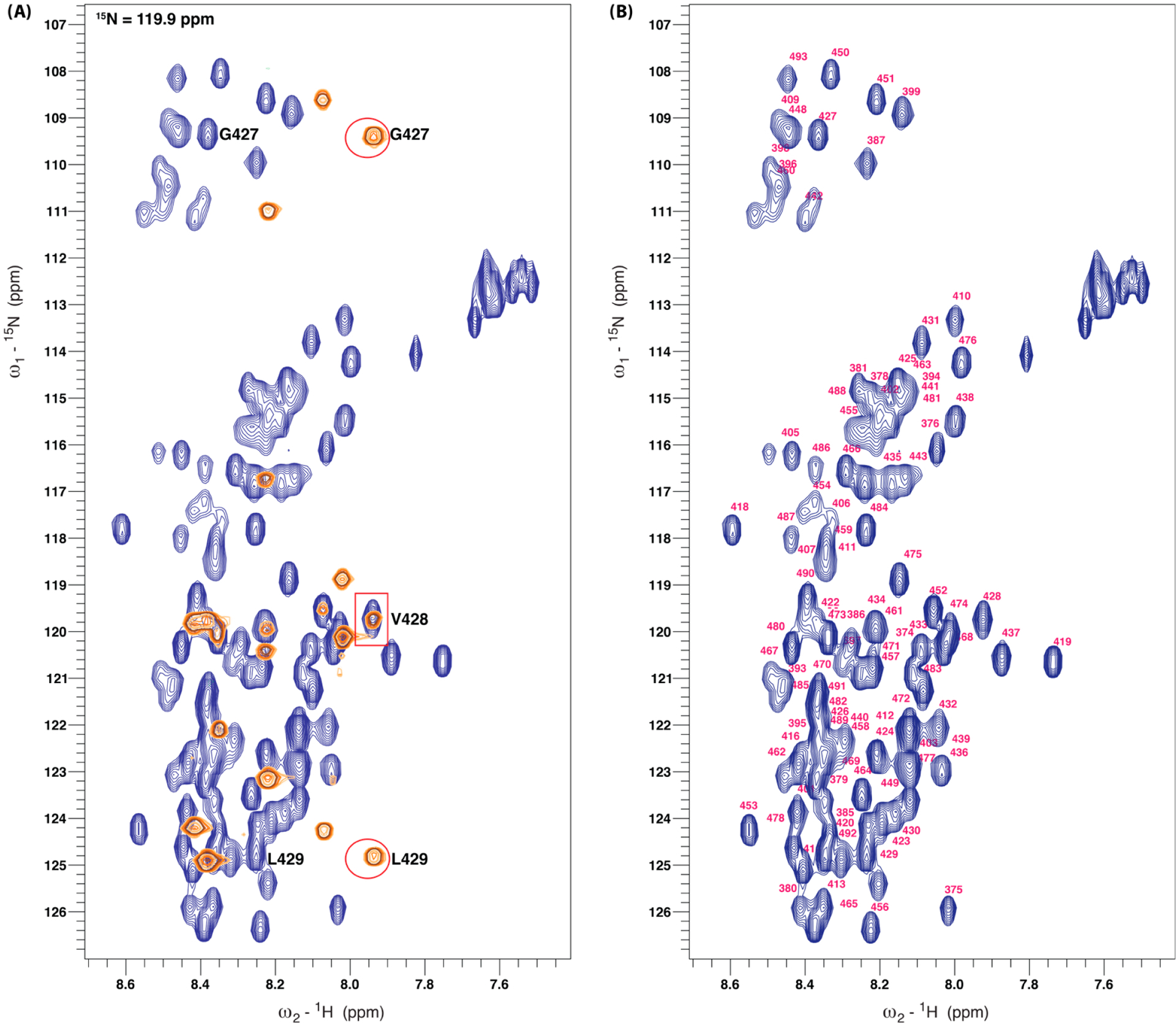
Assignment of LIC2-C 375–492 using 3D backbone NMR and HNN experiments. (**A**) F3-F2 plane displaying ^1^H-^15^N correlations through the 3D HNN spectrum at the F1-^15^N chemical shift of 119.9 ppm (orange) superimposed on the 2D ^1^H-^15^N HSQC spectrum (blue). Off-diagonal peaks (circles) indicate the ^15^N chemical shifts of the preceding and succeeding residues relative to the diagonal peak (square; V428). (**B**) ^1^H-^15^N HSQC spectrum demonstrating peak assignment.

We confirmed our prediction [12] that the LIC2-C protein is disordered through the narrow peak dispersion observed in the ^1^H-^15^N HSQC spectrum (Figure 2). Because of the combination of overlapping peaks, including the disorder of the protein and the existence of numerous redundant charged regions, we incorporated an HNN experiment into our standard backbone experiments. This additional experiment offers orthogonal connectivity information. (Figure 2A). Thus, we were able to obtain a near-complete backbone assignment (93.3%) (Table 1; Figure 2B). The missing residues are either heavily overlapped or in a sequence-redundant region. We have deposited the backbone assignment for LIC2-C in the BMRB under the accession code 51890.

## 4. Chemical Shift Analysis

**Figure 3.**
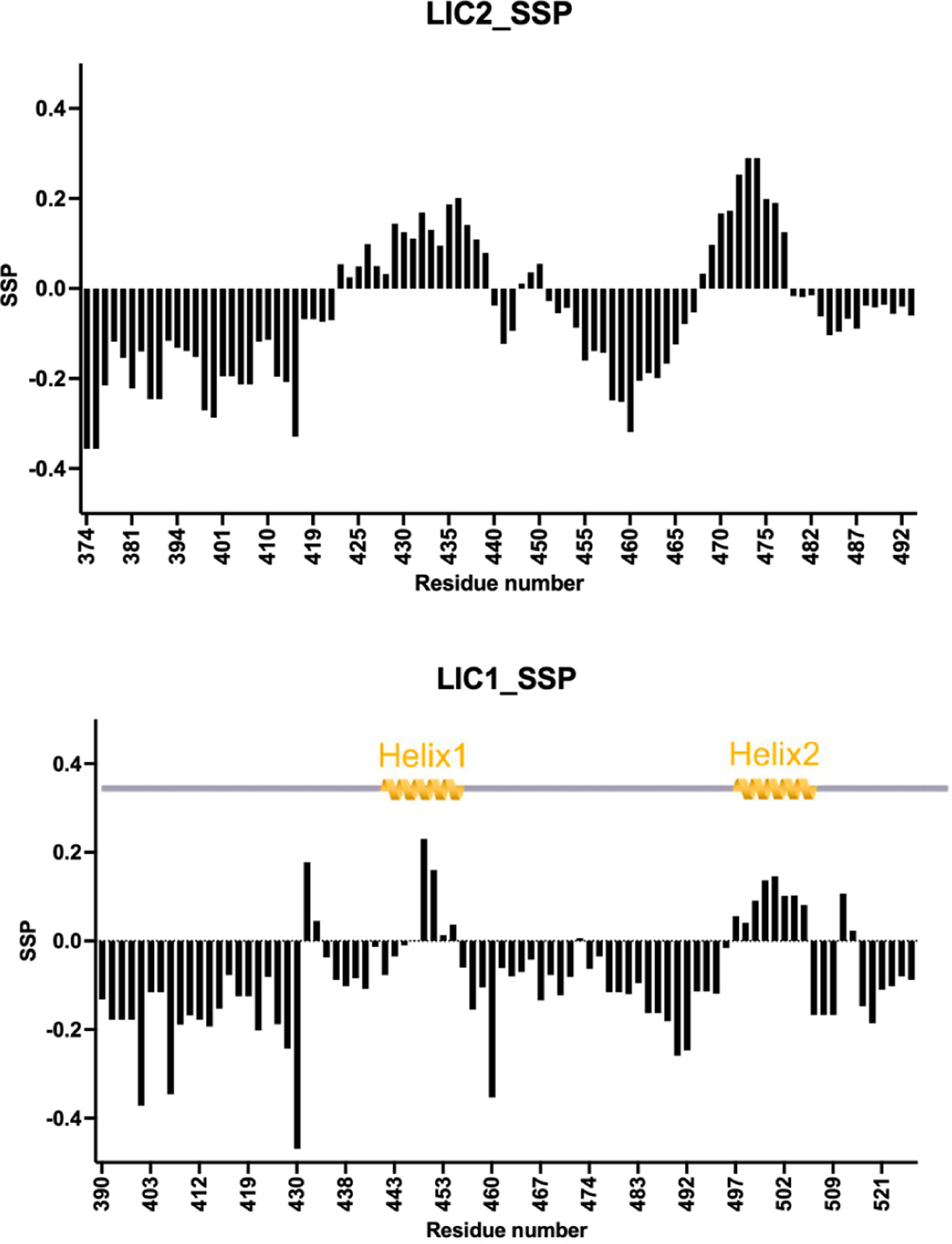
Secondary structure propensities for LIC2-C in comparison to LIC1-C. Data are derived from chemical shift values of ^1^HN, ^15^N, Cα, and Cβ. An SSP score of +1 indicates a fully formed α-helix, while −1 indicates a fully formed β-strand. Top: LIC2-C, regions 423–439 and 468–477 show helical propensity (helix1 and helix2) and are separated by more disordered parts. Bottom: LIC1-C, regions 442–453 and 493–502 have been shown to have helical propensity (helix 1 and helix2) [12].

We analyzed LIC2-C and compared it to LIC1-C by calculating the Secondary Structure Propensity (SSP) score [22] using HN, N, Cα, and Cβ chemical shifts. The SSP score varies between +1, representing a fully formed helix, and −1, indicating a fully formed β-strand. Generally, loops and disordered residues have an SSP score of around 0. The majority of the LIC2-C residues have a negative score, mostly more negative than −0.2 (Figure 3). This is indicative of structural disorder. However, we found two regions, residues 423–439 and 468–477, show a positive score, usually between 0 and +0.2, but peaking at ~0.3. These values are indicative of moderate helical propensity and, thus, of transiently formed helices. Interestingly these regions coincide with those that also show helical structure in LIC1 [12]. Of note, the scores for LIC2 are generally larger than for LIC1, suggesting a higher helical propensity. Following the nomenclature used for LIC1, we designate these LIC2 regions helix 1 (423–439) and helix 2 (468–477).

In LIC1, helix 1 constitutes the main interaction site for all tested dynein adaptors thus far and appears as a fully formed helix in dynein-dynactin-adaptor complexes in X-ray crystallography and cryo-electron microscopy images [23,24]. On the other hand, helix 2 is a secondary binding site for these adaptors except for Rab interacting lysosomal protein (RILP). Considering the conservation of both helix 1 and 2 in LIC2, it is reasonable to hypothesize that they may have a similar functional role in LIC2 as observed in LIC1. These findings are in agreement with our previous prediction of the presence of helical content, also based on the high amino acid conservation of these residues between LIC1 and LIC2 [12]. The results are also in line with our previous pulldown experiments, where we narrowed down the main adaptor interaction site to lie within residues 375 and 450, including L436 and L437.

The similarity of the helices stands in strong contrast to the fact that the phosphorylation sites on LIC1 are very different from those in LIC2 [25]. The C-terminal segment (LIC-C) is a hotspot for phosphorylation, and there is increasing evidence that LIC-adaptor interactions are spatiotemporally regulated by phosphorylation [26]. In contrast to the diverse range of kinesin transporters involved in plus-end directed intracellular transport, there is only one dynein available for many different types of transport, engaging many cargo-specific adaptors. Our results suggest that the multifunctionality of the dynein complex is neither facilitated by interaction sites that would be in different regions of LIC1 and LIC2, but more likely by post-translational modifications. The resonance assignment of LIC2, in combination with the previously published assignment of LIC1, serves as a foundation for future investigations into these unknown mechanisms.

## Figures and Tables

**Table 1.** Backbone assignment statistics of LIC2-C.

Construct	Total Number of Relevant Residues *	Total Number of Relevant Non-Proline Residues	% Backbone Resonances Assigned (Number of Assigned Backbone Atoms)
LIC2-C (LIC residues 375–492)	118	106	93.3% (99 ^15^N, 99 ^13^Cα, 87 ^13^Cβ)

* The construct has 14 additional non-relevant residues from cloning (preScission protease cleavage site; His-tag).

## Data Availability

The chemical shift assignment of the human LIC2-C (BMRB code 51890) has been deposited in the Biological Magnetic Resonance Data Bank.

## References

[1] Cianfrocco MA; Desantis ME; Leschziner AE; Reck-Peterson SL Mechanism and Regulation of Cytoplasmic Dynein. Annu. Rev. Cell Dev. Biol 2015, 31, 83. 26436706 10.1146/annurev-cellbio-100814-125438 PMC4644480

[2] Roberts AJ; Kon T; Knight PJ; Sutoh K; Burgess SA Functions and Mechanics of Dynein Motor Proteins. Nat Rev Mol Cell Biol 2013, 14, 713. 24064538 10.1038/nrm3667 PMC3972880

[3] Olenick MA; Holzbaur ELF Dynein Activators and Adaptors at a Glance. J. Cell Sci 2019, 132, jcs227132. 30877148 10.1242/jcs.227132 PMC6451413

[4] Reck-Peterson SL; Redwine WB; Vale RD; Carter AP The Cytoplasmic Dynein Transport Machinery and Its Many Cargoes. Nat. Rev. Mol. Cell Biol 2018, 19, 382. 29662141 10.1038/s41580-018-0004-3 PMC6457270

[5] Carter AP; Diamant AG; Urnavicius L How Dynein and Dynactin Transport Cargos: A Structural Perspective. Curr. Opin. Struct. Biol 2016, 37, 62–70. 26773477 10.1016/j.sbi.2015.12.003

[6] King SM Organization and Regulation of the Dynein Microtubule Motor. Cell Biol. Int 2003, 27, 213–215. 12681312 10.1016/s1065-6995(02)00337-2

[7] Mahale SP; Sharma A; Mylavarapu SVS Dynein Light Intermediate Chain 2 Facilitates the Metaphase to Anaphase Transition by Inactivating the Spindle Assembly Checkpoint. PLoS ONE 2016, 11, e0159646. 27441562 10.1371/journal.pone.0159646 PMC4956306

[8] Horgan CP; Hanscom SR; McCaffrey MW Dynein LIC1 Localizes to the Mitotic Spindle and Midbody and LIC2 Localizes to Spindle Poles during Cell Division. Cell Biol. Int 2011, 35, 171–178. 20964624 10.1042/CBI20100284

[9] Palmer KJ; Hughes H; Stephens DJ An InCytes from MBC Selection: Specificity of Cytoplasmic Dynein Subunits in Discrete Membrane-Trafficking Steps. Mol. Biol. Cell 2009, 20, 2885. 19386764 10.1091/mbc.E08-12-1160 PMC2695796

[10] Tynan SH; Purohit A; Doxsey SJ; Vallee RB Light Intermediate Chain 1 Defines a Functional Subfraction of Cytoplasmic Dynein Which Binds to Pericentrin. J. Biol. Chem 2000, 275, 32763–32768. 10893222 10.1074/jbc.M001536200

[11] Mahale S; Kumar M; Sharma A; Babu A; Ranjan S; Sachidanandan C; Mylavarapu SVS The Light Intermediate Chain 2 Subpopulation of Dynein Regulates Mitotic Spindle Orientation. Sci. Rep 2016, 6, 22. 28003657 10.1038/s41598-016-0030-3 PMC5431351

[12] Celestino R; Henen MA; Gama JB; Carvalho C; McCabe M; Barbosa DJ; Born A; Nichols PJ; Carvalho AX; Gassmann R; A Transient Helix in the Disordered Region of Dynein Light Intermediate Chain Links the Motor to Structurally Diverse Adaptors for Cargo Transport. PLoS Biol. 2019, 17, e3000100. 30615611 10.1371/journal.pbio.3000100 PMC6336354

[13] Even I; Reidenbach S; Schlechter T; Berns N; Herold R; Roth W; Krunic D; Riechmann V; Hofmann I DLIC1, but Not DLIC2, Is Upregulated in Colon Cancer and This Contributes to Proliferative Overgrowth and Migratory Characteristics of Cancer Cells. FEBS J. 2019, 286, 803–820. 30657258 10.1111/febs.14755

[14] Höing S; Yeh TY; Baumann M; Martinez NE; Habenberger P; Kremer L; Drexler HCA; Küchler P; Reinhardt P; Choidas A; Dynarrestin, a Novel Inhibitor of Cytoplasmic Dynein. Cell Chem. Biol 2018, 25, 357. 29396292 10.1016/j.chembiol.2017.12.014 PMC8543760

[15] Ikura M; Marion D; Kay LE; Shih H; Krinks M; Klee CB; Bax A Heteronuclear 3d NMR and Isotopic Labeling of Calmodulin: Towards the Complete Assignment of the ^1^H NMR Spectrum. Biochem. Pharmacol 1990, 40, 153–160. 2372304 10.1016/0006-2952(90)90190-v

[16] Cavanagh J Protein NMR Spectroscopy: Principles and Practice; Academic Press: Cambridge, MA, USA, 2007.

[17] Weisemann R; Ruterjans H; Bermel W 3D Triple-Resonance NMR Techniques for the Sequential Assignment of NH and 15N Resonances in 15N- and 13C-Labelled Proteins. J. Biomol. NMR 1993, 3, 113–120. 8448431 10.1007/BF00242479

[18] Hyberts SG; Milbradt AG; Wagner AB; Arthanari H; Wagner G Application of Iterative Soft Thresholding for Fast Reconstruction of NMR Data Non-Uniformly Sampled with Multidimensional Poisson Gap Scheduling. J. Biomol. NMR 2012, 52, 315–327. 22331404 10.1007/s10858-012-9611-z PMC3321367

[19] Delaglio F; Grzesiek S; Vuister GW; Zhu G; Pfeifer J; Bax A NMRPipe: A Multidimensional Spectral Processing System Based on UNIX Pipes. J. Biomol. NMR 1995, 6, 277–293. 8520220 10.1007/BF00197809

[20] Lee W; Tonelli M; Markley JL NMRFAM-SPARKY: Enhanced Software for Biomolecular NMR Spectroscopy. Bioinformatics 2015, 31, 1325–1327. 25505092 10.1093/bioinformatics/btu830 PMC4393527

[21] Vranken WF; Boucher W; Stevens TJ; Fogh RH; Pajon A; Llinas M; Ulrich EL; Markley JL; Ionides J; Laue ED The CCPN Data Model for NMR Spectroscopy: Development of a Software Pipeline. Proteins Struct. Funct. Genet 2005, 59, 687–696. 15815974 10.1002/prot.20449

[22] Marsh JA; Singh VK; Jia Z; Forman-Kay JD Sensitivity of Secondary Structure Propensities to Sequence Differences between α- and γ-Synuclein: Implications for Fibrillation. Protein Sci. 2006, 15, 2795–2804. 17088319 10.1110/ps.062465306 PMC2242444

[23] Lee IG; Olenick MA; Boczkowska M; Franzini-Armstrong C; Holzbaur ELF; Dominguez R A Conserved Interaction of the Dynein Light Intermediate Chain with Dynein-Dynactin Effectors Necessary for Processivity. Nat. Commun 2018, 9, 986. 29515126 10.1038/s41467-018-03412-8 PMC5841405

[24] Chaaban S; Carter AP Structure of Dynein–Dynactin on Microtubules Shows Tandem Adaptor Binding. Nature 2022, 610, 212–216. 36071160 10.1038/s41586-022-05186-y PMC7613678

[25] Kumari A; Kumar C; Wasnik N; Mylavarapu SVS Dynein Light Intermediate Chains as Pivotal Determinants of Dynein Multifunctionality. J. Cell Sci 2021, 134, jcs254870. 34014309 10.1242/jcs.254870

[26] Kumari A; Kumar C; Pergu R; Kumar M; Mahale SP; Wasnik N; Mylavarapu SVS Phosphorylation and Pin1 Binding to the Lic1 Subunit Selectively Regulate Mitotic Dynein Functions. J. Cell Biol 2021, 220, e202005184. 34709360 10.1083/jcb.202005184 PMC8562849

